# Supplementary figures and images for: The vegetation of Holocene coastal dunes of the Cape south coast, South Africa
Source: PeerJ. 2023 Dec 12;11:e16427. doi: 10.7717/peerj.16427 (PMC10722985; doi:10.7717/peerj.16427)

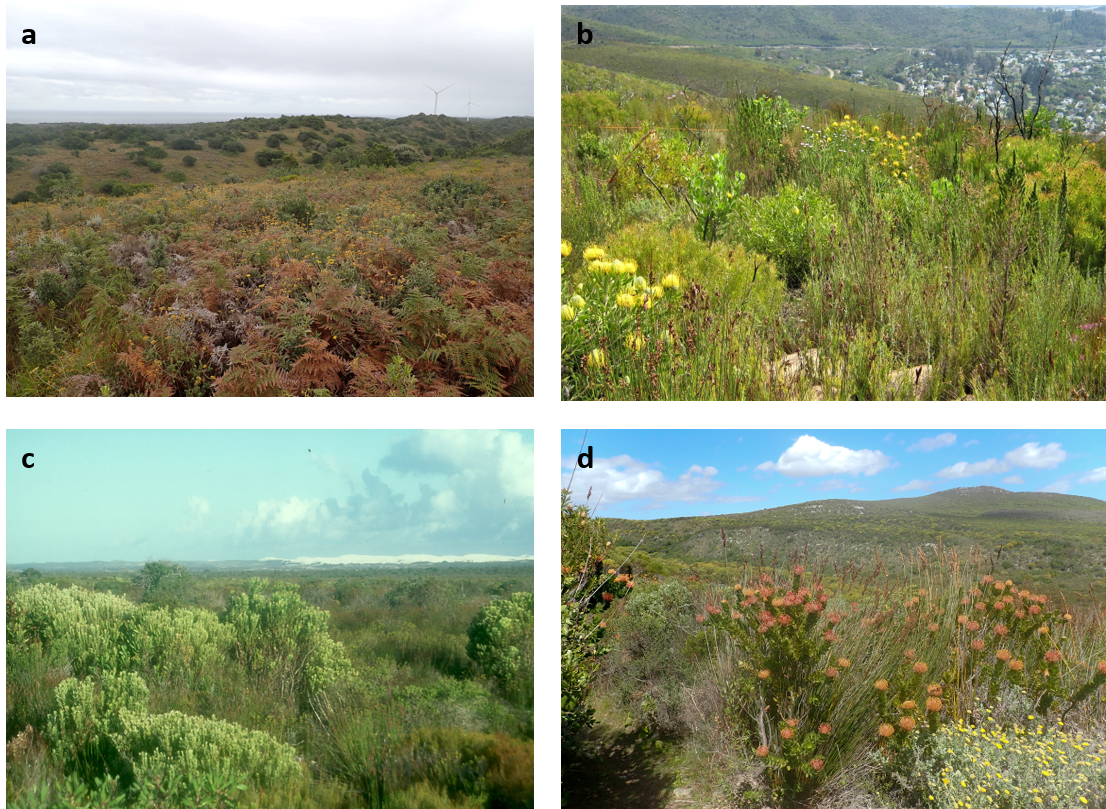

Supplement: Supplemental Information 3 — (A) Sand Fynbos on Nanaga (Neogene) sediments west of Oyster Bay. Dominant species are Pteridium aquilinum, Erica zeyheriana, Themeda triandra, Tristachya leocothrix and Thamnochortus glaber. Note the extensive Mesic Dune Thicket clumps on the inland-facing ridge in the background. (B) Sand Fynbos on Late Pleistocene sands (Waenhuiskrans Formation) near Knysna. Dominant species are Leucadendron salignum, Leucospermum cuneiforme, Passerina corymbosa, Anthospermum aethiopicum and Thamnochortus glaber. (C) Limestone Fynbos on Late Pleistocene aeolianite (Waenhuiskrans Formation) at De Hoop Nature Reserve. Dominant species are Leucadendron meridianum, Protea obtusifolia, Erica propinqua, Adenandra obtusata and Thamnochortus spp. (D) Sand Fynbos on Late Pleistocene sands (Waenhuiskrans Formation) near Gansbaai. Dominant species are Leucospermum patersonii, Leucadendron coniferum, Thamnochortus insignis and Helichrysum dasyanthemum. Photo credits: Richard Cowling (A,C,D); Antonella Tillard (B). [file peerj-11-16427-s003.png]

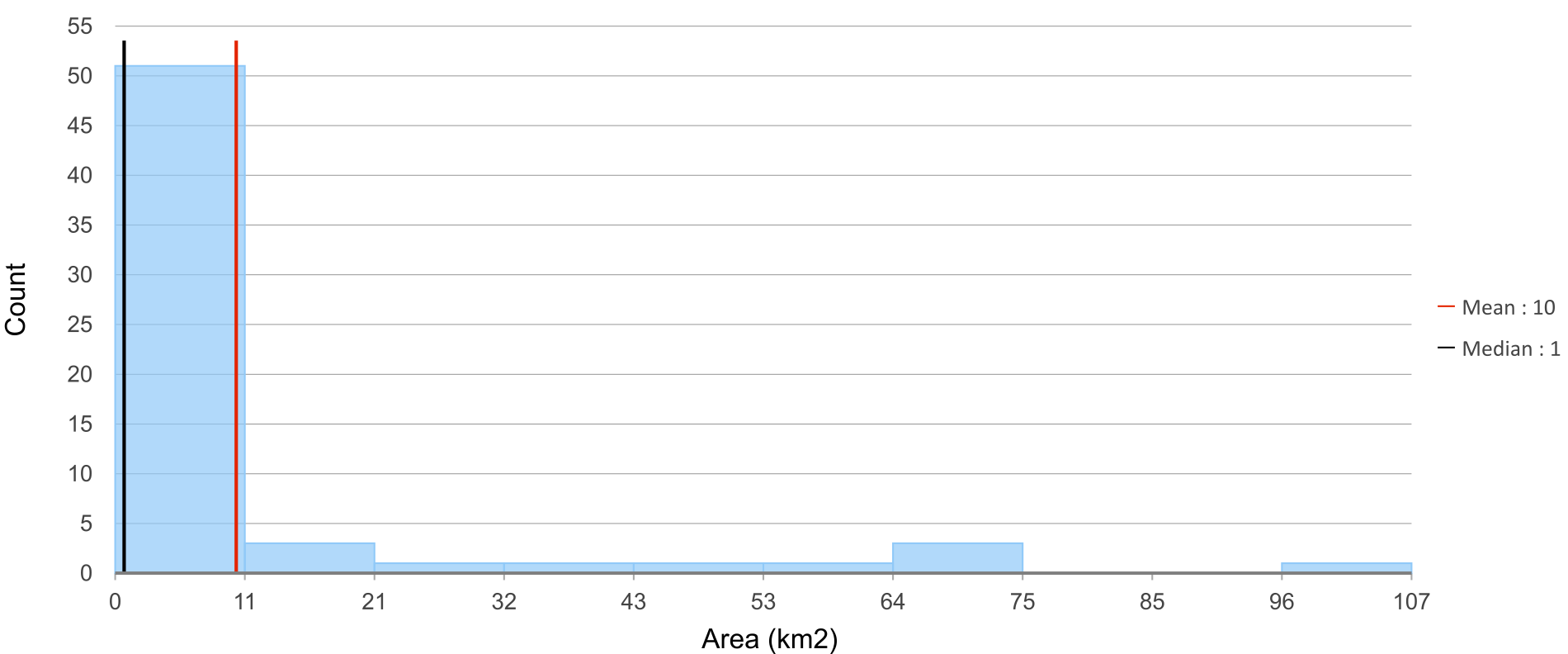

Supplement: Supplemental Information 5 — Most patches occupy <10 km2, with the distribution of surface area highly skewed towards patches covering ca. 1 km2. [file peerj-11-16427-s005.png]
